# Exploring the evidence behind the comparable impact of the pneumococcal conjugate vaccines PHiD-CV and PCV13 on overall pneumococcal disease

**DOI:** 10.1080/21645515.2021.1872341

**Published:** 2021-02-19

**Authors:** Patricia Izurieta, Javier Nieto Guevara

**Affiliations:** a GSK, Wavre, Belgium; b GSK, Panama City, Panama

**Keywords:** Pneumococcal conjugate vaccine, PHiD-CV, PCV10, PCV13, children, invasive pneumococcal disease, pneumonia, otitis media, serotypes, replacement

## Abstract

The worldwide implementation of pneumococcal conjugate vaccines (PCVs) in children has reduced the overall pneumococcal disease burden. Two PCVs are widely available for infant vaccination: the pneumococcal non-typeable *Haemophilus influenzae* protein D conjugate vaccine (PHiD-CV) and the 13-valent PCV (PCV13). While these PCVs differ in serotype composition (PCV13 includes polysaccharides of serotypes 3, 6A and 19A; PHiD-CV does not), their impact on the overall pneumococcal disease burden in children is comparable. This commentary summarizes the evidence of comparability between PHiD-CV and PCV13 and explores why differences in serotype composition may not necessarily translate into a differential clinical impact. Both vaccines confer similarly high protection against disease caused by vaccine serotypes and lead to a partial replacement by non-vaccine serotypes. PHiD-CV does not protect against serotype 3 disease (not included in the vaccine) and PCV13’s effect on this serotype has been inconsistent. PHiD-CV provides some cross-protection against disease caused by vaccine-related serotype 19A but neither vaccine has fully controlled 19A disease. While protection against 19A is higher for PCV13 than PHiD-CV, replacement by non-PCV13 serotypes in settings with a PCV13 program appears to compensate for this difference. This results in a similar residual overall disease burden with both vaccines.

## PLAIN LANGUAGE SUMMARY

What is the context?

- The pneumococcus bacterium can cause infections of the meninges, blood, lung, middle ear and sinuses.
- Two vaccins, *Synflorix* (GSK) and *Prevnar* 13 (Pfizer Inc.), are widely used to protect young children against these infections.
- The vaccines’ compositions differ: *Synflorix* includes antigens from 10 pneumococcus strains (or “serotypes”) and *Prevnar* 13 from 13 serotypes.
- However, both have a similar effect on the total pneumococcal disease burden in children.

What does this commentary highlight?

- This commentary summarizes the evidence beihnd the two vaccines’ comparable impact on pneumococcal disase.
- It also looks at why the vaccines have a similar effect on the total pneumococcal disease burden despite their different compositions.

What is the impact on current thinking?

- Given that *Synflorix* and *Prevnar* 13 have a comparable impact on pneumococcal disease, a country’s choice between the two vaccines will depend on vaccine supply, cost, logistical factors (e.g., transport, storage, training requirements of health workers) and the local pneumococcal epidemiology.

## Graphical abstract

**Figure:**
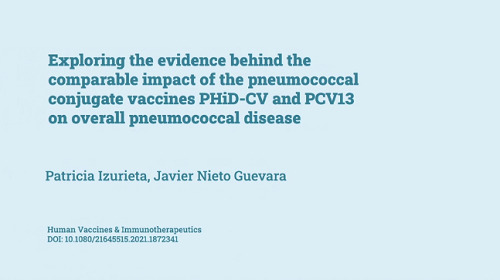
Video Abstract Read the transcript Watch the video on Vimeo © 2021 GlaxoSmithKline Biologicals SA. Published with license by Taylor & Francis Group, LLC

## Introduction

Before the widespread use of effective pediatric pneumococcal vaccines, *Streptococcus pneumoniae* killed >800,000 children aged 1–59 months worldwide each year (based on estimates for 2000).^1^ These deaths resulted from an estimated 14.5 million cases of invasive pneumococcal disease (IPD; including bacteremic pneumonia, meningitis and sepsis) and non-bacteremic pneumonia.^1^ While these numbers declined substantially after the worldwide implementation of pneumococcal conjugate vaccines (PCVs) in children, the latest estimates (from 2015) still show a significant burden: 9.2 million cases and >300,000 deaths per year, nearly 10% of all deaths in children aged 1–59 months.^2^ *S. pneumoniae* is also one of the main bacterial pathogens responsible for upper respiratory tract infections, including sinusitis and otitis media (OM).^3^

One hundred different pneumococcal serotypes have been identified, characterized by serologically distinct polysaccharide (PS) capsules.^4^ While most serotypes can cause disease, and significant variations in their distribution are seen by geographical region, time, age and disease manifestation, a small proportion of serotypes are responsible for most IPD in young children.^3,5–8^ Serotypes causing non-bacteremic pneumonia and OM are less well characterized as these diseases are primarily diagnosed through clinical assessment. The first PCV to enter the market – the seven-valent PCV (PCV7, *Prevnar/Prevenar*, Pfizer Inc.), in 2000 – contained PS of the seven serotypes that were most prevalent in pediatric IPD isolates in the United States: 4, 6B, 9V, 14, 18C, 19F and 23F.^5,7^ Higher-valent PCVs were subsequently developed, with serotype compositions that provided better global coverage and included serotypes that emerged after PCV7 introduction.^5,8^ Currently, two PCVs are widely available for vaccination in infants and young children: pneumococcal non-typeable *Haemophilus influenzae* (NTHi) protein D conjugate vaccine (PHiD-CV, *Synflorix*, GSK) and 13-valent PCV (PCV13, *Prevnar 13/Prevenar 13*, Pfizer Inc.). PHiD-CV contains PS from serotypes 1, 5 and 7F in addition to the seven PCV7 serotypes; PCV13 contains PS from the ten PHiD-CV serotypes and 3, 6A and 19A.^3,9^ PHiD-CV and PCV13 also differ in the amount of PS they contain for each serotype (more than double in PCV13 for seven of the ten serotypes shared with PHiD-CV, exceptions being 4, 18C and 19F), the carrier proteins conjugated to the PS (NTHi protein D for eight serotypes, tetanus and diphtheria toxoids for the remaining two in PHiD-CV; diphtheria toxoid variant CRM_197_ in PCV13) and the conjugation method.^9,10^ Despite these differences, the available evidence does not indicate that the two vaccines differ in their impact on the overall pneumococcal disease burden in children (i.e., the combined burden of disease caused by vaccine serotypes [VT] and non-VT).^3^

In this commentary, we summarize the evidence behind the comparable impact of PHiD-CV and PCV13 on pneumococcal disease, with an emphasis on the overall rather than VT-specific disease burden, as this is most relevant for public health. We explore why differences in serotype composition may not necessarily translate into a differential clinical impact. We focus on the vaccines’ effect on IPD (as this is the most frequently measured and etiologically characterized pneumococcal disease outcome) but also include a section describing their impact on pneumonia and OM.

## Comparable impact of PHiD-CV and PCV13 on overall IPD

The impact of PHiD-CV and PCV13 on IPD was evaluated in comprehensive systematic reviews. A systematic review of PCV effectiveness and impact studies in Latin America showed that both PHiD-CV and PCV13 have a significant impact on reducing IPD-related hospitalizations in <5-y-olds, with no evidence of one vaccine being superior over the other.^11^ Another systematic review (“PCV review of impact evidence [PRIME]”) evaluated both vaccines’ immunogenicity, and their effectiveness/impact on nasopharyngeal carriage, pneumonia, IPD and mortality.^9,12^ The review served as a basis for the latest update of the World Health Organization (WHO) opinion paper on PCVs in <5-y-olds, which states that, while differences were found in the impact on the three additional PCV13 serotypes and 6C, there is at present insufficient evidence of a difference in the net impact of the two products on the overall pneumococcal disease burden (from IPD and pneumonia).^3^

**Figure 1.**
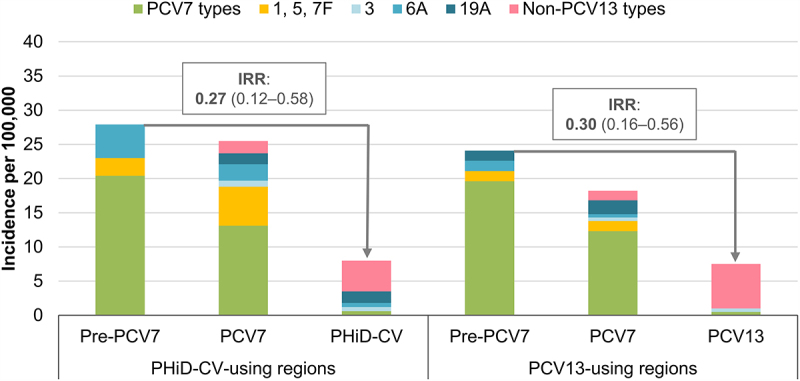
Invasive pneumococcal disease incidence rates in children 0–2 y of age in PHiD-CV- or PCV13-using Swedish regions before PCV7 and before and after PHiD-CV or PCV13 implementation.

IRR, incidence rate ratio; PCV13, 13-valent pneumococcal conjugate vaccine; PCV7, 7-valent pneumococcal conjugate vaccine; PHiD-CV, pneumococcal non-typeable *Haemophilus influenzae* protein D conjugate vaccine. Data from Naucler et al.^13^ IRRs (with 95% confidence intervals) compare the incidences of overall invasive pneumococcal disease after PHiD-CV or PCV13 implementation with those before PCV7 implementation.

Both reviews based their conclusions on indirect comparisons between the two vaccines, since no prospective, randomized, head-to-head studies assessing the effects of PHiD-CV and PCV13 on pneumococcal diseases have been performed. However, a single study in Sweden has been published since, that compared the vaccines when given to equivalent populations in parallel time periods and similar settings, which is the closest available to a head-to-head study.^13^ In Sweden, PCV7 was introduced in some regions from 2007 and throughout the country in 2009. It was replaced by PHiD-CV in some and PCV13 in other regions from 2009 onwards.^13^ Declines in overall IPD (IPD caused by any serotype) in 0–2-y-old children were not statistically significantly different between PHiD-CV- and PCV13-using regions (thereby confirming the overall conclusions of the systematic reviews): incidence rate ratios (IRRs) compared to the pre-PCV7 period were 0.27 (95% confidence interval [CI]: 0.12–0.58) and 0.30 (0.16–0.56), respectively (Figure 1).^13^

While this study is – to our knowledge – the only one comparing the effects of the two vaccines on IPD when used in the same time period and the same country, other countries/regions used both vaccines consecutively in their infant immunization programs, providing an opportunity to evaluate the vaccines in similar settings and populations (but different time periods). We found a single study in such settings (in Quebec [Canada]) that calculated the effectiveness against IPD for both vaccines (see Supplementary material for details on the literature search).^14^ In Quebec, PCV7 was introduced for routine infant immunization in 2004 and replaced by PHiD-CV in 2009. PHiD-CV was replaced by PCV13 in 2011^14^ and was reinstated in 2018.^15^ A case-control study assessing laboratory-confirmed IPD cases from 2–59-month-old children, notified during 2005–2013, showed comparable effectiveness of PHiD-CV and PCV13 against overall IPD: 72% (95% CI: 46–85) and 66% (29–83) for ≥1 dose of the vaccines, respectively.^14^

## Why can PHiD-CV and PCV13 have a comparable impact on overall IPD?

As highlighted in the introduction, PCVs only cover a small fraction of all pneumococcal serotypes.^9^ Undeniably, the direct effectiveness against VT disease and the relative contribution of VT to the overall disease burden in the population targeted for vaccination are the major components determining the maximal vaccine effect. The net impact can be increased if the vaccine confers “cross-protection” against non-VT that serologically resemble one of the VT (i.e., vaccine-related types).^16^ By contrast, the net impact on disease can be diminished as a result of “serotype replacement”, a consequence of prolonged PCV use, where VT are replaced by non-VT in nasopharyngeal carriage and disease.^17^

In the following sections, we discuss how the combined effect on VT, vaccine-related serotypes and non-VT can result in a similar impact of PHiD-CV and PCV13 on overall IPD in children. We also briefly discuss herd protection.

### Protection against VT IPD

**Figure 2.**
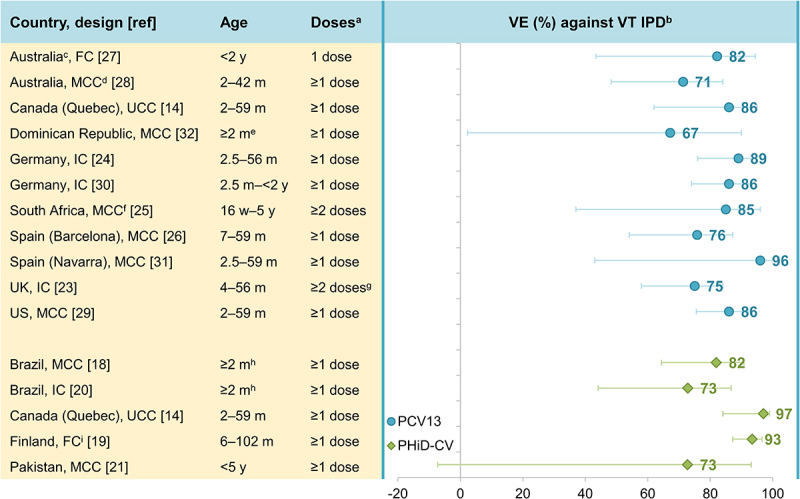
Effectiveness of PCV13 and PHiD-CV against VT IPD in children.

We evaluated data from effectiveness studies to assess whether PHiD-CV and PCV13 protect equally well against VT IPD and if protection is equally high for each serotype (see Supplementary material for details on the literature search and Supplementary table 1 for study details). Effectiveness studies using case-control, indirect or full cohort designs have shown statistically significant effectiveness against VT IPD: 73–97% for PHiD-CV (against PHiD-CV types)^14,18–22^ and 67%–96% for PCV13 (against PCV13 types) (Figure 2).^14,22–32^ This is in line with results from two double-blind, randomized, controlled PHiD-CV trials showing efficacy/effectiveness estimates of 92–100% against VT IPD.^33,34^ No randomized efficacy trials have been performed with PCV13 in children.

FC, full cohort; IC, indirect cohort; m, months of age; MCC, matched case-control; PCV13, 13-valent pneumococcal conjugate vaccine; PHiD-CV, pneumococcal non-typeable *Haemophilus influenzae* protein D conjugate vaccine; UCC, unmatched case-control; UK, United Kingdom (England, Wales and Northern Ireland in^23^); US, United States; VE, vaccine effectiveness; VT IPD, vaccine-type invasive pneumococcal disease (see note ^b^ below); w, weeks of age; y, years of age. Error bars indicate 95% confidence or credible intervals (CIs). ^a^Effectiveness estimates are for the indicated number of doses. Estimates for ≥1 dose were used where available. ^b^For PCV13 effectiveness estimates: IPD caused by PCV13 serotypes 1, 3, 4, 5, 6A, 6B, 7F, 9V, 14, 18C, 19A, 19F, 23F and – for references^23,24^ – vaccine-related 6C. For PHiD-CV estimates: IPD caused by PHiD-CV serotypes 1, 4, 5, 6B, 7F, 9V, 14, 18C, 19F, 23F and – for reference^14^ – vaccine-related 6A. ^c^Non-Aboriginal children only. ^d^Two different designs were used in this study to estimate VE against VT IPD: MCC: values in graph, and IC: VE was 76% (95% CI: 46–89). ^e^Interquartile range for age of cases: 5.3–17.4 m, and controls: 4.8–15.3 m. ^f^Children not infected with human immunodeficiency virus only. ^g^ ≥2 doses before 12 m or 1 dose on or after 12 m. ^h^Actual age of included children was 2.6–53.1 m. ^i^Three different designs were used in this study: FC: values in graph, MCC: VE against VT IPD was 98% (95% CI: 90–100), and IC: VE against VT IPD was 100% (95% CI: 98–100).

**Figure 3.**
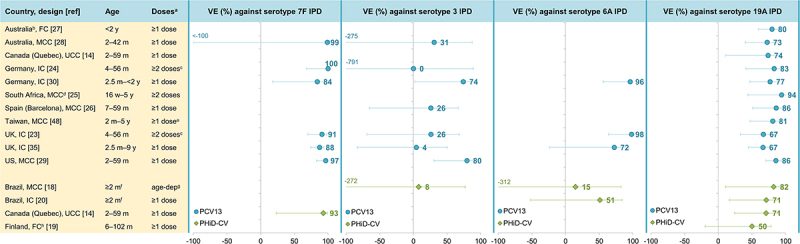
Effectiveness of PCV13 and PHiD-CV against serotype-specific IPD in children.

The question of whether PCVs protect equally well against each VT is harder to address because the number of IPD cases included in individual studies is often too low to calculate serotype-specific effectiveness. Nevertheless, effectiveness estimates are available from several studies for serotypes 1, 3 and 7F, as well as for 6A and 19A (covered in the next section). Effectiveness against 7F IPD was high across studies for both PCVs: 93% for PHiD-CV^14^ and 84–100% for PCV13 (Figure 3).^23,24,28–30,35^ Effectiveness estimates against serotype 1 IPD were only available for PCV13 and were also high across most studies (83–89%),^23,25,26,30^ although a lower estimate was found in a UK study (66%).^35^ By contrast, no consistent effect of PCV13 on IPD due to serotype 3 (included in PCV13, not in PHiD-CV) has been demonstrated, in line with the immunologic hyporesponsiveness observed for this serotype.^36,37^ Most studies showed no statistically significant effectiveness against serotype 3 IPD, and estimates varied widely across studies (0–80%) (Figure 3).^23,24,26,28–30,35^ As expected, given that PHiD-CV does not contain serotype 3, PHiD-CV has no effect on serotype 3 IPD.^12,18^ Consistent with these findings, serotype 3 is still present as a cause of pediatric IPD in PHiD-CV- and PCV13-using countries,^12,13,38–41^ and is frequently associated with PCV13 vaccine failure.^13,42,43^ Serotype-specific effectiveness estimates for PHiD-CV and PCV13 against IPD caused by the seven VT contained in PCV7 are available from a limited number of studies^18,20,26,28^ because in most settings, PCV7 had been used for several years before higher-valent PCV introduction, resulting in too low numbers of PCV7-type IPD cases to calculate serotype-specific effectiveness.

FC, full cohort; IC, indirect cohort; IPD, invasive pneumococcal disease; m, months of age; MCC, matched case-control; PCV13, 13-valent pneumococcal conjugate vaccine; PHiD-CV, pneumococcal non-typeable *Haemophilus influenzae* protein D conjugate vaccine; UCC, unmatched case-control; UK, United Kingdom (England, Wales and Northern Ireland for^23^ and England for^35^); US, United States; VE, vaccine effectiveness; w, weeks of age; y, years of age. Error bars indicate 95% confidence or credible intervals (CIs). ^a^Effectiveness estimates are for the indicated number of doses. Estimates for ≥1 dose were used where available. ^b^Non-Aboriginal children only. ^c^ ≥2 doses before 12 m or 1 dose on or after 12 m. ^d^Children not infected with human immunodeficiency virus only. ^e^ ≥1 dose with the first dose given before 2 y. ^f^Actual age of included children was 2.6–53.1 m. ^g^Number of doses was age-dependent: up to date for age for the recommended number of PHiD-CV doses. ^h^Three different designs were used in this study: FC (values in graph), MCC: VE against 19A IPD was 45% (95% CI: −54–79) and IC: VE against 19A IPD was 66% (95% CI: −76–95).

In summary, while both vaccines are highly effective against VT IPD, protection is not equally high for all serotypes; in particular, PCV13 does not seem effective against serotype 3 IPD in most settings where the vaccine was evaluated.^23,24,26,28,35^

### (Cross-)protection against IPD caused by serotypes 6A, 6C and 19A

Data on the cross-protection of PHiD-CV against 6A IPD (mediated by cross-reactive 6B antibodies^44^) are scarce given the low baseline levels of 6A, particularly in countries with a previous PCV7 program (due to PCV7 cross-protection against 6A disease).^12,16^ Case-control and indirect cohort studies in Brazil (which introduced PHiD-CV as first PCV in its infant immunization program) did not show significant effectiveness of PHiD-CV against 6A IPD (Figure 3).^18,20^ However, population-based cohort studies in Finland (which also introduced PHiD-CV as first PCV) noted a significant and sustained reduction in 6A IPD in vaccine-eligible children over a 3- and 6-y follow-up, suggesting long-lasting direct cross-protection against 6A IPD.^45,46^ In Sweden, 6A IPD declined (non-significantly) in PHiD-CV-using regions (IRR versus PCV7 period in 0–2-y-old children: 0.23, 95% CI: 0.02–2.24) (Figure 1).^13^ For PCV13, effectiveness estimates against 6A IPD were 72–98% across the assessed studies but not all estimates were statistically significant because of the low baseline 6A IPD rates (Figure 3).^23,30,35^

Evidence of PCV13 cross-protection against 6C IPD (mediated by cross-reactive 6A antibodies^47^) is sparse although positive effectiveness estimates were found in an indirect cohort study in the UK (70%, 95% CI: 2–92)^35^ and a case-control study in Australia (80%, <−100–98).^28^ PHiD-CV effectiveness against 6C IPD was not assessed in the evaluated studies.^14,18–20^

Cross-protection has been observed for PHiD-CV against 19A IPD (mediated by cross-reactive 19F antibodies).^44^ Significant effectiveness against 19A IPD was shown in two studies in Brazil using either a case-control or indirect cohort design (82% and 71%) and in a case-control study in Quebec (71%), with the latter showing similar effectiveness for PCV13 (74%) (Figure 3).^14,18,20^ Positive but non-significant effectiveness was also observed for PHiD-CV in a study in Finland (50%, full cohort design).^19^ Population-based studies in Finland showed a significant reduction (62%, 95% CI: 20–85) in 19A IPD in a vaccine-eligible cohort after a 3-y follow-up post-PHiD-CV implementation,^45^ but this reduction was no longer significant after a 6-y follow-up (26%, −13–51).^46^ Possible explanations suggested by the authors were waning of the initial cross-protection afforded by PHiD-CV or an increasing infection pressure from 19A in Finland during the later follow-up years.^46^ In Sweden, no reduction in the incidence of 19A IPD was seen in 0–2-y-olds in PHiD-CV-using regions (versus the PCV7 period), while, over the same time period, the 19A IPD incidence decreased significantly in PCV13-using regions (no cases identified in the PCV13 period) (Figure 1).^13^ Interestingly, while effectiveness estimates for PCV13 against 19A IPD (67–94%)^14,23–30,35,48^ tend to be higher than those for PHiD-CV, they were lower than those against 7F and 6A measured in the same studies (Figure 3),^23,24,28–30,35,49^ suggesting that PCV13 may be less effective against 19A than against other VT. The seemingly lower effectiveness of both vaccines against 19A IPD is consistent with the observation that 19A is still circulating in various settings several years after these vaccines were introduced,^38–41,50,51^ and with the observation that 19A is among the most common VT responsible for PCV13 vaccine failure.^42,43^

In summary, PHiD-CV provides some cross-protection against 19A, but neither PHiD-CV nor PCV13 has fully controlled 19A IPD. For 6A and 6C, data are too limited to draw firm conclusions, but some of the available evidence is suggestive of cross-protection by PHiD-CV against 6A and by PCV13 against 6C.

### Replacement by non-VT

Previous experience with PCV7 showed a complete replacement of VT by non-VT in the nasopharynx after vaccine introduction, but an incomplete replacement in disease.^17,38,52^ Consequently, PCV7 had no net impact on pneumococcal carriage but its net impact on pneumococcal disease was substantial in many settings. This seeming discrepancy can be explained by a lower virulence of the replacing non-VT compared to VT.^17,38^ Similar observations have been made for PHiD-CV and PCV13, with both vaccines leading to serotype replacement in carriage^53–57^ and disease.^38–40^

We found two published, randomized, controlled trials that assessed the impact of both vaccines on carriage (see Supplementary material for search string).^58,59^ No major differences were found in either study between the carriage prevalence of VT, non-VT or any pneumococcal serotype in PHiD-CV- compared to PCV13-vaccinated children.^58,59^

In terms of IPD, as mentioned previously, no randomized, head-to-head studies are available but the study in Sweden (the closest available to a head-to-head comparison) showed that the incidence of IPD due to non-PCV13 serotypes in 0–2-y-olds increased non-significantly in PHiD-CV-using regions (IRR compared to the PCV7 period: 2.48, 95% CI: 0.51–12.0) and significantly in PCV13-using regions (IRR compared to the PCV7 period: 4.78, 1.22–18.7) (Figure 1).^13^ The residual IPD burden in 0–2-y-olds in Sweden was very similar in PHiD-CV- and PCV13-using regions but the share taken up by 6A and 19A in regions using PHiD-CV was taken up by non-PCV13 serotypes in those using PCV13 (Figure 1). Therefore, considering the redistribution of serotypes, there were no differences in the impact of these PCVs on overall IPD in this age group.^13^ Previous reviews of surveillance studies and reports showed replacement of VT by non-VT in disease in both PHiD-CV- and PCV13-using countries but did not allow direct comparisons between the two vaccines.^38–40^

In summary, both PHiD-CV and PCV13 cause serotype replacement. At present, there is insufficient evidence to conclude whether replacement is more pronounced with one vaccine or the other.

### Herd protection

The total benefit of PCV programs does not only depend on direct effects on the vaccinated population but also on indirect (herd) effects on unvaccinated populations. VT IPD and nasopharyngeal carriage have declined in various age groups of unvaccinated children and adults after implementation of both vaccines.^9,12^ The study in Sweden, comparing PHiD-CV- and PCV13-using regions, showed similar indirect effects on overall IPD with both vaccines.^13^

## Comparable impact of PHiD-CV and PCV13 on pneumonia and OM

As for IPD, no prospective, head-to-head studies directly comparing the effect of PHiD-CV and PCV13 on pneumonia or OM have been performed. The systematic review on effectiveness and impact studies in Latin America and the PRIME systematic review found evidence of both PCVs’ impact on clinical and chest X-ray confirmed pneumonia in young children and concluded that there was no systematic evidence that one product has a greater impact than the other on pneumonia.^9,11,12^ Time-series analyses of national-level mortality data from Latin American and Caribbean countries showed declines in mortality due to all-cause pneumonia in countries using either vaccine.^60^

Two studies in Sweden compared OM-related endpoints in PHiD-CV- and PCV13-using regions.^61,62^ A first study retrospectively analyzed national registry data and showed that the incidence of outpatient acute OM (AOM), AOM hospital admissions, myringotomies and ventilation tube insertions declined in <5-y-olds both after PHiD-CV and PCV13 introduction.^61^ The decrease in outpatient AOM and ventilation tube insertions appeared more pronounced in PHiD-CV-using regions; however, cautious interpretation is warranted because geographical differences in incidence were large before vaccine introduction.^61^ The second study, a retrospective cohort study using linked regional and national databases, assessed the OM incidence in one PHiD-CV- and one PCV13-using region. Descriptive time-series analyses showed declines in the OM incidence in ≤2-y-olds after both PHiD-CV and PCV13 implementation. Effectiveness estimates obtained by modeling adjusted for age, period and cohort effects were only statistically significant for PHiD-CV in ≤5-y-olds.^62^

In summary, both vaccines seem to have a comparable impact on pneumonia and although some studies suggest that PHiD-CV may offer better protection against OM-related endpoints, further evidence is necessary to confirm these findings.

## Limitations

Our commentary was not intended as an exhaustive review of the impact of both PCVs but rather as an overview of their comparability. It was therefore based on evidence generated in previous systematic reviews providing indirect comparisons, complemented by studies where both vaccines were assessed in the same settings. We did not perform a comprehensive systematic search to retrieve these studies and may therefore have missed some of the available evidence. Claims of comparability of the two vaccines in terms of effectiveness against pneumococcal diseases are limited by the lack of prospective, randomized, head-to-head studies. Indirect comparisons between studies are hindered by differences in surveillance methods, clinical practice, local epidemiology, PCV vaccination schedule, previous PCV use and duration of the PCV programs. Our analysis of serotype-specific effectiveness is hampered by the fact that data for some of the serotypes are sparse or absent, particularly for PHiD-CV.

## Conclusions

Studies with different designs assessing various pneumococcal-related endpoints in different settings have shown that PHiD-CV and PCV13 have a comparable impact on the overall pneumococcal disease burden. For IPD, this comparability can be explained by the similarly high effectiveness of both vaccines against VT disease, the limited effect of PCV13 on serotype 3 disease and PHiD-CV’s observed cross-protection against 19A (and possibly 6A) disease. While the effect of PHiD-CV on 19A is lower than that of PCV13, neither vaccine has fully controlled 19A IPD. Moreover, the comparative study in Sweden has shown that, in PCV13-using settings, 19A (and 6A) are replaced by disease-causing non-PCV13 serotypes, hence resulting in a comparable residual IPD burden. Both PCVs have also shown similar clinical outcomes for OM and pneumonia.

Considering the comparable impact of both vaccines on the overall pneumococcal disease burden (despite their different compositions), product choice will ultimately depend on programmatic characteristics, vaccine supply, price and local pneumococcal epidemiology.^3^

## Supplementary Material

Supplemental Material

## Trademark statement

*Synflorix* is a trademark of the GSK group of companies. *Prevnar/Prevenar* and *Prevnar 13/Prevenar 13* are trademarks of Pfizer Inc.
