# Supplementary material for: Exploring the evidence behind the comparable impact of the pneumococcal conjugate vaccines PHiD-CV and PCV13 on overall pneumococcal disease
Source: Hum Vaccin Immunother. 2021 Feb 19;18(1):1872341. doi: 10.1080/21645515.2021.1872341 (PMC8920200; doi:10.1080/21645515.2021.1872341)
Supplement: Supplemental Material [file KHVI_A_1872341_SM6673.docx]

**Supplementary material**

**Exploring the evidence behind the comparable impact of the pneumococcal conjugate vaccines PHiD-CV and PCV13 on overall pneumococcal disease**

*By Patricia Izurieta et al.*

**Literature searches**

The effectiveness studies included in our commentary (sections “Comparable impact of PHiD-CV and PCV13 on overall IPD” and “Why can PHiD-CV and PCV13 have a comparable impact on overall IPD?”) were retrieved from a systematic review by Berman-Rosa et al.,^1^ complemented by a literature search. The systematic review included papers published until July 2018. To find studies published after this date, we searched PubMed for articles on observational studies assessing the effectiveness of pneumococcal non-typeable *Haemophilus influenzae* protein D conjugate vaccine (PHiD-CV) or 13-valent pneumococcal conjugate vaccine (PCV13) against invasive pneumococcal disease (IPD), by using the following search string: (("pneumococcal conjugate vaccine" OR PCV10 OR PHiD-CV OR PCV13 OR 10-valent OR 13-valent) AND ("effectiveness")) AND (("2018/01/01"[Date - Entry] : "3000"[Date - Entry])). The search was performed in August 2020 and retrieved 153 articles. Titles and abstracts were screened to identify observational effectiveness studies (using a case control, indirect or full cohort design) on PHiD-CV or PCV13 against IPD in children. Five articles were included in addition to the twelve retrieved from the Berman-Rosa review (**Supplementary table 1**).

The two head-to-head carriage studies were identified through a literature search in PubMed, using the following search string: (PCV10 OR PHiD-CV OR 10-valent) AND (PCV13 OR 13-valent) AND (carriage OR colonization), without limits on publication dates and using “randomized controlled trial” as filter.

**Supplementary table 1. Characteristics of effectiveness studies**

| **Ref** | **Country** | **Study design** | **Vaccine (year of introduction in immunization program^a^)** | **Schedule during study period** | **Study period^b^** | **Comments on study methodology** |
| --- | --- | --- | --- | --- | --- | --- |
| ^2^ | Australia | Full cohort | PCV13 (2011)  Prior PCV7 (2005) | 3+0 | 2008–2013^c^ | No adjustments for confounders required |
| ^3^ | Australia | Matched case-control  Indirect cohort | PCV13 (2011)  Prior PCV7 (2005) | 3+0 | 2011–2014 | Case-control: matched by date of birth, indigenous status and jurisdiction of residence.  Indirect cohort: VE adjusted for age |
| ^4^ | Brazil | Matched case-control | PHiD-CV (2010) | 3+1 | 2010–2012 | Matched by age and neighborhood  VE adjusted for receipt of at least 1 dose of DTP-Hib vaccine and any chronic illness |
| ^5^ | Brazil | Indirect cohort | PHiD-CV (2010) | 3+1 | 2010–2012 | VE adjusted for date of admission/medical attention, age at illness, day care attendance and receipt of at least 1 dose of DTP |
| ^6^ | Canada (Quebec) | Unmatched case-control | PCV13 (2011)  PHiD-CV (2009)  Prior PCV7 (2004) | 2+1 | 2010–2013 | VE adjusted for age, year, season and underlying medical conditions including asthma and severe prematurity |
| ^7^ | Dominican Republic | Matched case-control | PCV13 (2013) | 2+1 | 2013–2016 | Matched by age and neighborhood of residence, adjustment for low weight-for-age Z score (malnutrition) and home built of wood (socioeconomic proxy) |
| ^8^ | Finland | Full cohort  Nested case-control  Indirect cohort | PHiD-CV (2010) | 2+1 | 2010–2018 | Full cohort: VE adjusted for age, sex and calendar year  Nested case-control: matched by age, sex and calendar year  Indirect cohort: VE adjusted for age, sex and calendar year |
| ^9^ | Germany | Indirect cohort | PCV13 (2009)^d^  Prior PCV7 (2006) | 3+1 | 2010–2014 | VE adjusted for year of infection and age (except for VE against 7F IPD, which was calculated without these adjustments) |
| ^10^ | Germany | Indirect cohort | PCV13 (2009)^d^  Prior PCV7 (2006) | 3+1 | 2010–2015 | VE adjusted for season and age |
| ^11^ | Pakistan | Matched case-control | PHiD-CV (2013) | 3+0 | 2013–2017 | Matched by age, district and season  VE adjusted for sex, age, paternal education, use of natural gas for cooking, crowding and exposure to smoke based on propensity scores |
| ^12^ | South Africa | Matched case-control | PCV13 (2011)  Prior PCV7 (2009) | 2+1 | 2012–2014 | Matched by age, surveillance site and HIV status  VE adjusted for malnutrition, receipt of 3 doses of DTP vaccine at 16 weeks of age and maternal education level |
| ^13^ | Spain (Barcelona) | Matched case-control | PCV13 (not included in publicly funded immunization program during study period) | 3+1 | 2012–2016 | Matched by hospital, age, sex, date of hospitalization and underlying medical condition  VE adjusted for confounders |
| ^14^ | Spain (Navarra) | Matched case-control | PCV13 (not included in publicly funded immunization program during study period) | 3+1 | 2010–2014 | Matched by pediatric practice, district of residence and date of birth  VE adjusted for sex and parental income level |
| ^15^ | Taiwan | Matched case-control | PCV13 (2015, catch-up from 2013) | 3+1 | 2007–2013 | Matched by age, gender and residence  VE adjusted for DTP and influenza vaccination history |
| ^16^ | UK (England, Wales and Northern Ireland) | Indirect cohort | PCV13 (2010)  Prior PCV7 (2006) | 2+1 | 2010–2013 | VE adjusted for year of infection and age |
| ^17^ | UK (England) | Indirect cohort | PCV13 (2010)  Prior PCV7 (2006) | 2+1 | 2006–2018 | VE adjusted for year of infection and age |
| ^18^ | US | Matched case-control | PCV13 (2010)  Prior PCV7 (2000) | 3+1 | 2010–2014 | Matched by age and postal code  No adjustment for confounders |

DTP(-Hib), diphtheria-tetanus-pertussis(-*Haemophilus influenzae* type b) vaccine; HIV, human immunodeficiency virus; IPD, invasive pneumococcal disease; PCV13, 13-valent pneumococcal conjugate vaccine; PCV7, 7-valent pneumococcal conjugate vaccine; PHiD-CV, pneumococcal non-typeable *Haemophilus influenzae* protein D conjugate vaccine; VE, vaccine effectiveness

^a^Year of introduction refers to the year the specified vaccine was introduced in the publicly funded universal infant immunization program of the country or specified region. In several countries, the vaccines were available prior to these dates, either publicly funded for risk groups or on the private market.

^b^Study period for PHiD-CV or PCV13 evaluation is indicated where specified in the original articles.

^c^PCV13 effectiveness evaluated in children born from 2008 to include children who received a supplementary PCV13 dose, which was offered between 10/2011 and 9/2012 to infants born from 11/2008 to 10/2010 who had completed their primary PCV7 course.

^d^In Germany, PHiD-CV is available but the vast majority of children receive PCV13.

**References**

1. Berman-Rosa M, O'Donnell S, Barker M, Quach C. Efficacy and effectiveness of the PCV-10 and PCV-13 vaccines against invasive pneumococcal disease. Pediatrics. 2020;145(4):e20190377. doi: 10.1542/peds.2019-0377.

2. Gidding HF, McCallum L, Fathima P, Moore HC, Snelling TL, Blyth CC, Jayasinghe S, Giele C, de Klerk N, Andrews RM, et al. Effectiveness of a 3 + 0 pneumococcal conjugate vaccine schedule against invasive pneumococcal disease among a birth cohort of 1.4 million children in Australia. Vaccine. 2018;36(19):2650-2656. doi: 10.1016/j.vaccine.2018.03.058.

3. Jayasinghe S, Chiu C, Quinn H, Menzies R, Gilmour R, McIntyre P. Effectiveness of 7- and 13-valent pneumococcal conjugate vaccines in a schedule without a booster dose: a 10-year observational study. Clin Infect Dis. 2018;67(3):367-374. doi: 10.1093/cid/ciy129.

4. Domingues CM, Verani JR, Montenegro Renoiner EI, de Cunto Brandileone MC, Flannery B, de Oliveira LH, Santos JB, de Moraes JC. Effectiveness of ten-valent pneumococcal conjugate vaccine against invasive pneumococcal disease in Brazil: a matched case-control study. Lancet Respir Med. 2014;2(6):464-471. doi: 10.1016/s2213-2600(14)70060-8.

5. Verani JR, Domingues CM, de Moraes JC, Brazilian Pneumococcal Conjugate Vaccine Effectiveness Study Group. Indirect cohort analysis of 10-valent pneumococcal conjugate vaccine effectiveness against vaccine-type and vaccine-related invasive pneumococcal disease. Vaccine. 2015;33(46):6145-6148. doi: 10.1016/j.vaccine.2015.10.007.

6. Deceuninck G, De Serres G, Boulianne N, Lefebvre B, De Wals P. Effectiveness of three pneumococcal conjugate vaccines to prevent invasive pneumococcal disease in Quebec, Canada. Vaccine. 2015;33(23):2684-2689. doi: 10.1016/j.vaccine.2015.04.005.

7. Tomczyk S, Lessa FC, Sánchez J, Peña C, Fernández J, Gloria Carvalho M, Pimenta F, Cedano D, Whitney CG, Verani JR, et al. Effectiveness of 13-pneumococcal conjugate vaccine (PCV13) against invasive pneumococcal disease in children in the Dominican Republic. BMC Infect Dis. 2018;18(1):152. doi: 10.1186/s12879-018-3047-3.

8. Rinta-Kokko H, Auranen K, Toropainen M, Nuorti JP, Nohynek H, Siira L, Palmu AA. Effectiveness of 10-valent pneumococcal conjugate vaccine estimated with three parallel study designs among vaccine-eligible children in Finland. Vaccine. 2020;38(6):1559-1564. doi: 10.1016/j.vaccine.2019.11.049.

9. Weinberger R, van der Linden M, Imohl M, von Kries R. Vaccine effectiveness of PCV13 in a 3+1 vaccination schedule. Vaccine. 2016;34(18):2062-2065. doi: 10.1016/j.vaccine.2016.02.043.

10. van der Linden M, Falkenhorst G, Perniciaro S, Fitzner C, Imohl M. Effectiveness of pneumococcal conjugate vaccines (PCV7 and PCV13) against invasive pneumococcal disease among children under two years of age in Germany. PLoS One. 2016;11(8):e0161257. doi: 10.1371/journal.pone.0161257.

11. Riaz A, Mohiuddin S, Husain S, Yousafzai MT, Sajid M, Kabir F, Rehman NU, Mirza W, Salam B, Nadeem N, et al. Effectiveness of 10-valent pneumococcal conjugate vaccine against vaccine-type invasive pneumococcal disease in Pakistan. Int J Infect Dis. 2019;80:28-33. doi: 10.1016/j.ijid.2018.12.007.

12. Cohen C, von Mollendorf C, de Gouveia L, Lengana S, Meiring S, Quan V, Nguweneza A, Moore DP, Reubenson G, Moshe M, et al. Effectiveness of the 13-valent pneumococcal conjugate vaccine against invasive pneumococcal disease in South African children: a case-control study. Lancet Glob Health. 2017;5(3):e359-e369. doi: 10.1016/s2214-109x(17)30043-8.

13. Dominguez A, Ciruela P, Hernandez S, Garcia-Garcia JJ, Soldevila N, Izquierdo C, Moraga-Llop F, Diaz A, de Sevilla MF, Gonzalez-Peris S, et al. Effectiveness of the 13-valent pneumococcal conjugate vaccine in preventing invasive pneumococcal disease in children aged 7-59 months. A matched case-control study. PLoS One. 2017;12(8):e0183191. doi: 10.1371/journal.pone.0183191.

14. Guevara M, Barricarte A, Torroba L, Herranz M, Gil-Setas A, Gil F, Bernaola E, Ezpeleta C, Castilla J, Working Group for Surveillance of the Pneumococcal Invasive Disease in Navarra. Direct, indirect and total effects of 13-valent pneumococcal conjugate vaccination on invasive pneumococcal disease in children in Navarra, Spain, 2001 to 2014: cohort and case-control study. Euro Surveill. 2016;21(14). doi: 10.2807/1560-7917.Es.2016.21.14.30186.

15. Su WJ, Lo HY, Chang CH, Chang LY, Chiu CH, Lee PI, Lu CY, Hsieh YC, Lai MS, Lin TY. Effectiveness of pneumococcal conjugate vaccines of different valences against invasive pneumococcal disease among children in Taiwan: a nationwide study. Pediatr Infect Dis J. 2016;35(4):e124-133. doi: 10.1097/inf.0000000000001054.

16. Andrews NJ, Waight PA, Burbidge P, Pearce E, Roalfe L, Zancolli M, Slack M, Ladhani SN, Miller E, Goldblatt D. Serotype-specific effectiveness and correlates of protection for the 13-valent pneumococcal conjugate vaccine: a postlicensure indirect cohort study. Lancet Infect Dis. 2014;14(9):839-846. doi: 10.1016/s1473-3099(14)70822-9.

17. Andrews N, Kent A, Amin-Chowdhury Z, Sheppard C, Fry N, Ramsay M, Ladhani SN. Effectiveness of the seven-valent and thirteen-valent pneumococcal conjugate vaccines in England: The indirect cohort design, 2006-2018. Vaccine. 2019;37(32):4491-4498. doi: 10.1016/j.vaccine.2019.06.071.

18. Moore MR, Link-Gelles R, Schaffner W, Lynfield R, Holtzman C, Harrison LH, Zansky SM, Rosen JB, Reingold A, Scherzinger K, et al. Effectiveness of 13-valent pneumococcal conjugate vaccine for prevention of invasive pneumococcal disease in children in the USA: a matched case-control study. Lancet Respir Med. 2016;4(5):399-406. doi: 10.1016/s2213-2600(16)00052-7.
